# Mr. Ward, on Opium

**Published:** 1804-06-01

**Authors:** M. Ward

**Affiliations:** Manchester


### *To the Editors of the Medical and Physical Journal.*

Gentlemen,

NEARLY five years have elapsed since I first ventured to call in question the propriety of attempting to exhibit medicines *internally* in hydrophobia and tetanus, and more than two since I took the liberty a second time of protesting against the continuance of this most injudicious practice; or I should rather have said, *this ingenious mode of tormenting*;* but so imperfect was my knowledge of the true nature of the diseases at that time, that my objections were founded more on the horror expressed by this unfortunate

* See Medical and Physical Journal, vol. i. p. 447-9; vol. vi. p. 478-480.

tunate class of patients (invariably in hydrophobia and very often in tetanus) at the sight of either food or medicine, and on the *uniform failure* of this method of treatment during a period of at least *two thousand years*, (a length of time much more than sufficient to shew that the plan was altogether improper) than on any correct ideas I could pretend to have formed respecting them; and the more I reflected on the subject, the stronger was my conviction of the necessity of *an entire change in our manner of proceeding*, before any progress could be made in the methodus medendi. Upon this ground it was that I recommended the external application of opium, as well as because it seemed well calculated by its property of diminishing morbid irritability, to counteract the most dangerous and distressing symptom (the spasmodic affection of the muscles, particularly those employed in deglutition) we have to encounter in these "*most dreadful of human disorders*:" But the plan I proposed has not, to my knowledge, been tried in hydrophobia;* and only in five cases of tetanus that I have heard of†, in some of which it was evidently useful, though its effects were not so decisive as I expected; indeed, in one case only has it been fairly tried, and in that it succeeded completely.‡ But long before this happened, my opinion had been, that our want of success in the cure of these maladies was more owing to the want of a systematic arrangement of the phenomena, and the judicious application of the knowledge to which such an arrangement would lead, than to any

---

* Opium was applied externally in a case of hydrophobia that occurred in this town last summer; but cantharides and opium were given internally at the same time: electricity was also employed. As usual, the case terminated fatally.

† Of five cases of tetanus in which the opiate frictions were used, *four were cured*, but in all of them other means were employed at the same time, which renders it difficult to say precisely, what share of the merit was due to the former. Can it be shewn however, that an equal degree of success has attended the treatment of the same, or any given number of cases taken in succession, *where internal remedies alone were employed?* And does it not hold out a strong inducement to relinquish the use of internal remedies in hydrophobia; at least, until a rational theory of the disease shall have been formed, capable of directing us in the choice of more appropriate remedies than those in present use?

Frictions with opium and mercury are said to have been employed with advantage in tetanus two or three years ago, by Mr. Mursinna, at Berlin.—*Med. Journ.*

‡ Med. and Phys. Journal, vol. 6, p. 433.

any other cause; and these ideas took such firm hold of my mind as to have led me to reflect, frequently and earnestly, on the ætiology and symptomatology of the diseases in question, and am inclined to hope some benefit may accrue to this department of medical science from my labours; indeed, I am greatly deceived if I have not found out a path which will, *if hydrophobia be remediable*, eventually lead to success. At any rate I have discovered (which is an acquisition of no small import, as it must certainly lead the way to a plan of proceeding totally different to those in common use, the necessity of which I imagine will be obvious) *why medicines administered internally, never did,* NOR NEVER WILL, SUCCEED *in subduing it.**

I shall not at present enter into a detail of the method I have pursued in prosecuting my inquiries, or the data on which my conclusions are founded: These, with a few remarks on the pathology of diabetes, and some farther observations on opium, may probably be published at my leisure, should the reception of the following be such as to encourage me to proceed.

The design of this paper is merely to announce to the Medical World, through the medium of your Journal, *the result of my inquiries,* (as some time may probably elapse before the whole will be ready for public inspection;) in doing which I shall purposely avoid entering into any reasoning on the subject, farther than is necessary to render what I have to say intelligible, and to enable your readers to reduce my ideas to practice, should they be inclined to think favourably of them. All I beg is, that they will not hastily condemn a scheme of practice, which appears to me, on the most mature deliberation, better calculated than any hitherto proposed, to subdue *the most terrific malady to which human nature is liable, and which has continued its ravages uncontrouled, from the earliest accounts we have of it, to the present time.*

Without farther preface, I shall proceed to state the principal conclusions at which I have arrived.

1. Hydrophobia and tetanus belong to the same natural class and order.†

2. The

* Undoubtedly, patients afflicted with *tetanus* do now and then struggle through the disease, though treated in the common way; but so seldom, as only to form an exception to a general rule.

† Dr. Cullen's classification appears to be correct, *as far as it goes;* but that of Dr. Darwin is preferable on some accounts.

2. The remote cause varies according as the disease is idiopathic or symptomatic.

3. The predisposing cause consists in *an unequal and irregular distribution of the nervous or sensorial power*; the tendency to which is increased by external heat, debility in the muscular and other coats of the stomach and intestines, and of the involuntary muscles generally, impure air, &c. &c.

4. The proximate cause consists in *an exuberance and retrograde action of the faculties of the sensorium affecting the voluntary muscles.**

5. The proximate effect is the disease itself, which consists in *a spasmodic and retrograde motion of the fibres of the voluntary muscles.*

6. *In violent tetanus every voluntary muscle in the body is affected, as described in No. 5.*

7. *In hydrophobia, the spasmodic and retrograde motions specified in No. 5, are principally confined to the pharynx, the œsophagus, the larynx, the epiglottis, the tongue, and the muscles employed in deglutition;* (the stomach, though an *involuntary* muscle, is often affected in the same manner), *hence the characteristic symptom of the disease, horror at the approach of liquids or food; hence also the inefficacy and fatal consequences of administering medicines internally, and the cruelty of urging the patient to swallow liquids, &c.*† *There are also convulsive motions of the heart and arteries, evinced*

---

* There are some who refuse their assent to the doctrine of a retrograde action of the vessels altogether, though it is a fact as well established as any in Anatomy or Medicine; others there are, who admit that it may take place in the muscular coats of the stomach and intestines, but deny the possibility of its occurrence in the absorbents, on account of the valves being so numerous and strong as to support a column of mercury when injected after death; which, they say, must necessarily, and at all times, prevent a regurgitation of their contents; not considering that the valves are composed of muscular fibres and vessels as well as the absorbents, and that when the action of the absorbents is inverted, the action of the valves must be inverted also.

† To be convinced of the truth of this reasoning, we have only to turn our attention to *the relative situation* of the parts above mentioned, and to keep in mind *their natural action*, particularly that of the tongue and epiglottis, in the act of swallowing, and compare it with *the spasmodic and retrograde action* with which they are affected, and which render swallowing so difficult and dangerous in hydrophobia. We must also keep in mind the vicinity of the œsophagus and trachea, their similarity of form, the intimate connection that subsists between them in the offices they perform, and how apt parts so circumstanced are to take on the same kind of action; the sphincter vesicæ et ani, for example.

*evinced by the violent palpitations which often take place: At the same time the voluntary muscles belonging to the chest and extremities are variously and violently agitated and convulsed,* (the nervous power in them being abundant, and its action retrograde, but less so than in spasm; the energy of the brain seems in some cases to be also increased:) *in some instances there is merely an increased action of the voluntary muscles;* in others the latter are affected, partially or generally, with spasmodic or retrograde action as in tetanus; all these circumstances contributing to produce that wonderful and horrible variety observable in the disease.*

Of the other symptoms, it will be sufficient to observe at present, that the principal ones, such as the lassitude, *the shooting pains preceding the attack of hydrophobia, from the part bitten upwards towards the head or heart, and never in a contrary direction,* the great depression of spirits, restlessness, *extreme sensibility to all impressions, the violent and long continued efforts to vomit* †, *the sense of suffocation, thirst, delirium; the vehement and incessant exertions to get rid of the saliva, the variable state of the pulse and respiration, &c. &c.* will be easily and fully explained on the principles laid down in No. 3, 4, and 5; and those which are yet to be brought forward.

The poison insinuated into a wound from the bite of a rabid animal, appears to exert its influence *principally if not entirely upon the nervous system;* though the size and depth of the wound, and the violence used in inflicting it, causing the skin and muscles to be more or less lacerated, seems to have some effect both in producing the disease, and in determining the duration of the interval between the bite and the accession of the disease. A good deal may also depend on the nervous system of the patient being more or less irritable.

What is the precise state of the inoculated part in the interval between the healing of the wound and the coming on of the hydrophobia; and how the poison operates in producing those changes in it which immediately precede the disease, are points which have not been explained; but I hope to be excused if I say, I do not consider them altogether inexplicable.

8. At

* As in the case described by Mr. John Hunter, where the patient was relieved by running round Smithfield. See Heads of Inquiry in the Transactions of a Medical Society, vol. i.

† See Hamilton on Hydrophobia, vol. i. p. 213—219.

8. At the same time that certain parts of the system are affected as described in No. 6 and 7, there is *a deficiency* of the nervous or sensorial power in the *involuntary* muscles, *which is in proportion to the exuberance in the voluntary*; hence the vital and natural functions are carried on in a weak and inefficient manner, from the sensibility, irritability, and mobility; or in one word, the *contractility* of the parts employed in carrying on these functions, (namely the heart, arteries, muscular coats of the stomach and intestines) being greatly diminished. *And this is the reason why tetanic patients bear such enormous quantities of opium and wine, without experiencing the usual effects.** It also shews why opium *taken internally*, inasmuch as it tends to increase this *want of contractility* in the muscular fibres of the alimentary canal, both by rendering them incapable of receiving, and the nerves which supply them of transmitting, the necessary supply of nervous power, must be injurious; *by lessening action where it ought to be increased*, namely, in the stomach, intestines, heart, and arteries; *and increasing it where it ought to be diminished*, namely, in the *voluntary* muscles.†

Thus it appears, that three different states of the nervous power subsist *at the same time; namely, an exuberance, a deficiency, and a retrograde action. The voluntary muscles are the seat of the first and third, and the involuntary of the second;* but whether the deficiency in the latter be the cause or the effect of the exuberance in the former, I shall not at present undertake to determine. I suspect however, it is sometimes the *cause*, sometimes the *effect* of the exuberance, and the consequent inordinate increase of the animal functions.

Another circumstance which constitutes *a leading feature* of hydrophobia and tetanus, and which will assist in explaining the variety already noticed, is, the great propensity in the nervous power *suddenly to shift its situation*, in such a manner as to cause *an exuberance* where there was *a deficiency* just before, and vice versa. This is clearly evinced by the sudden sensation of the convulsions or spasms in the voluntary muscles, and their being immediately succeeded by *violent palpitations of the heart, vomiting,*

---

* An insensibility to the action of opium frequently occurs in mania, and from a similar cause.

† This remark is, in general, more applicable to tetanus than to hydrophobia.

*ing, or retching; or by severe spasms about the throat; or by convulsive, spasmodic, or retrograde action in some of the other organs:* but it seldom, if ever, happens, that *all* these symptoms are present *at the same time;* and for this plain reason, that whenever there is *an abundance* of the nervous power in one set of muscles, there is *a proportionable deficiency* in another set.

Suffocation is the most frequent cause of death happening so suddenly in these diseases, particularly in hydrophobia, from the spasmodic and retrograde action of the pharynx and œsophagus extending to the larynx, trachea, intercostal and other muscles of respiration, *so as to put an immediate and entire stop to inspiration.* But in many instances the patient falls back and dies instantly, on attempting to swallow either food or medicine; in which case a part of it probably enters the larynx and trachea, causing instant suffocation; owing to the tongue, and consequently the epiglottis, *being forced forwards,* when the glottis is of course left unguarded.

Another frequent cause of death, especially in tetanus, is, *the great monopoly and expenditure of the nervous power by the voluntary muscles, from the long continuance of the spasms; in consequence of which, the heart and large arteries do not receive a sufficient supply of this power to enable them to carry on their functions.*

9. The indications of cure, according to this view of the subject, will be clear and simple, viz. 1st. *to restore the balance in the distribution of the nervous or sensorial power;* and, 2dly, *the natural, that is, the* PROGRESSIVE *action of the muscular fibres.*

10. *No medicine, or combination of medicines, administered internally, can be adequate to the production of these effects.*

11. To restore the balance in the distribution of the n. p. which forms the first indication (No. 9) the superfluous or exuberant portion must be repelled *from the voluntary to the involuntary muscles,* (i. e. from the circumference to the centre) *which can only be done by some external application capable of giving a sudden shock to the system, so as to diminish the contractility of the fibres of the voluntary muscles, and also the mobility of the n. p.** *in the nerves distributed to the latter; to be repeated oftener or seldomer, according to the violence and frequency of the spasms, which will*

* See Cullen's Institutes of Medicine.

*will be in proportion to the greater or less tendency to a return of the circumstances mentioned in No. 3 and 4.*

12. *Cold water seems, a priori, well adapted to fulfil the ends proposed in No.* 11 *.

13. To restore the progressive action of the muscular fibres, which forms the second indication (No. 9), the water should be applied in such a manner as to pass over the body, *in a direction contrary to the morbid or retrograde motion of the fibres of the voluntary muscles; for which purpose, and also for that insisted on in No.* 10, *it should invariably be poured on the head and upper parts of the body,* (the patient being placed in an erect position supported by two assistants;) *but in every variety of the disease, the largest part, as well as the force used in applying it, should be principally directed to those parts of the body most affected with spasm:* for example, in hydrophobia or trismus, to the sides of the face, throat and neck; in opisthotonos joined with trismus, to the back, sides of the face and neck; and so in the other varieties. Two or three quarts, of a moderate temperature (perhaps about 40 of Fahrenheit) would be sufficient to begin with in an adult; gradually increasing the quantity to five or six gallons, and reducing the temperature, if that should be found expedient, as low as the freezing point; *care being taken to wipe the body dry with warm cloths immediately after, and to place it in bed between blankets till the warmth is restored, or the time returns for repeating the application* †. Should this treatment occasion too great a depression of the powers of life, bladders of hot water might be kept in readiness to apply to the stomach, and bottles of hot water or warm bricks to the feet.

14. *The*

* As a preparatory step to this treatment, and also to relieve the spasms about the fauces, cold water should be sprinkled on *the face, throat, and parts adjacent,* as often as the paroxysm returns. If this should be serviceable, the aspersion might be gradually extended to the chest, belly, and extremities, the body being wiped dry with warm cloths, and removed into warm blankets directly after. In many cases this will perhaps be as much as the patient will be prevailed upon to submit to.

† In a practice so new as is here recommended, and a disorder so unmanageable as hydrophobia, unforeseen circumstances may easily occur to defeat, not only these, but the most judicious plans art can devise; which renders it utterly impossible to lay down rules applicable to every case. It is highly probable this paper will be found to contain mistakes, both in theory and practice, as well as omissions, which it will be for experience to rectify and supply.

14. * *The patient should on no account be urged to take either food or drink,* MUCH LESS MEDICINE, *as long as any difficulty in swallowing remains; nor should fluids of any kind be agitated in his hearing: on the contrary, every thing should be studiously avoided which is likely to excite any uneasiness or apprehension; for which reason he should be desired to ask, or if unable to speak, to express his wish by a sign, whenever he feels an inclination for refreshment.* At such times there would probably be no impropriety in allowing him to take his choice of such beverages as the following.

Lemonade; barley water sweetened with honey; gruel, or barley water, with wine, sugar, and spice; mulled wine, or good negus; beef tea, broth, &c: and a saline draught with lemon juice in the act of effervescence, once in two or three hours.

15. Principally to procure stools, but partly with a view to nourishment, glisters made of gruel, sugar, butter, and salt, should be injected every three or four hours, till the former effect is obtained †. Should they fail, and the patient be able to swallow with ease (which sometimes happens in tetanus) half an ounce of castor oil should be given in two or three spoonsfull of tartarized infusion of senna, or in any of the above mentioned liquids, every three or four hours, until the bowels are relaxed.

With a view to *the prevention of hydrophobia,* excision should *never* be omitted when the wounded part is so situated as to allow of it; and instead of limiting this operation to a few days after the accident, as is usually done, I should be inclined to perform it at any distance of time *under two years,* (the disease having appeared as late as nineteen months‡;) or even when the patient begins to complain, *provided the pain and discolouration be confined to the bitten part, and no dread of liquids has taken place.*

But where, either from a dislike to the knife, or the wound being so situated as not to admit of its use, this practice

* This caution is as necessary to be attended to in tetanus, *when the spasms extend to the parts contained in the throat and fauces,* as in hydrophobia.

† Two or three drachms of tincture of opium might be added to each glister, should the motions of the stomach be retrograde: but it sometimes happens the sphincter ani is strongly contracted, and the introduction of a pipe has the effect of bringing on the spasms, in which case they must of course be omitted.

‡ Trans. Med. Soc. V. 1, p. 304.—Ham. V. 2, p. 134.

practice is not adopted, the *pure water of kali* should be carefully and diligently applied, as soon after the accident as possible.

As a prophylactic, *a shower bath*, used two or three times a week for a few months, seems far preferable to bathing either in fresh or sea water, both for preserving that *equilibrium* in the distribution of the nervous power, which is so essential to health; and also for preventing a *retrograde action* of the vessels*: to which should be added, *gentle* exercise on horseback, a light nutritious diet, with the occasional use of such laxatives as increase the peristaltic motion of the intestines.

After the commencement of the disease, the wounded part being painful, and excision not having been performed, I see no impropriety in destroying the life of the part, by applying a pledget spread thick over with the calx cum kali puro, and a poultice afterwards.

The analogy subsisting between hydrophobia, tetanus, hysteria, and diabetes, leads me to believe, that a method of treatment somewhat similar to the above, may be of use in the latter. In the early stage of the disease, before the retrograde action of the absorbents is firmly established, I should expect great benefit from the judicious use of *a shower bath*, together with a saline draught in the act of effervescence, every four or five hours, and bitters and laxatives occasionally; joined with a diet consisting principally of animal food.

I have not time to enlarge on this subject now; but it may not be amiss to throw out a few hints and cautions, should any of your readers be inclined to adopt the plan.

If the disorder be recent, and the patient's strength not much reduced, it might be proper to begin with water of the heat of the atmosphere; but if of long standing, and the patient be weak and hectical, the temperature of the water should be at least 60 of Fahrenheit the first week of using it; and should be reduced a few degrees every week: but if at any time he complains of being shivery and weaker after using the bath, the water should be made a little warmer a few times afterwards, and should never be reduced so low, if it can be avoided, as to cause these inconveniences.

Secondly, As soon as the patient is able to bear it, the

* May not the good effects of cold affusion in fever be explained upon these principles?

cold affusion should be used *instead of the shower bath,* gradually increasing the quantity of water; and if this agrees, after using it once a day for a short time, and the progress towards recovery is slow, it might perhaps be applied twice a day with advantage: *but the temperature of the water, and the frequency of its application, should always be adapted to the feelings and strength of the patient, the state of the weather, &c.* The proper time for applying it will be in a morning, as soon as the patient rises, or mid-way between breakfast and dinner; and at five or six in the afternoon, should it ever become expedient to apply it twice a day.

Thirdly, The patient should be in *an erect posture* whenever the shower bath or the cold affusion is made use of, and should stand on a board raised and made a little convex, with grooves and perforations to prevent the water accumulating about his feet.

Fourthly, He should be rubbed dry with warm cloths, and wrapped in a warm blanket immediately after using the bath or affusion.

Simple and self-evident as some of these precautions may seem, they are of more consequence than at first sight may appear; indeed, the success of the scheme will depend in a great measure on the manner in which it is executed.

For instance, the cold affusion was lately recommended by me, in a case of diabetes in an elderly man, after every thing else had been tried in vain.

The temperature was to be reduced, five degrees daily, from 60 of Fahrenheit to the freezing point.

The first application felt grateful to the patient; the second, of 55, too cold; the third, of 50, still more so: he complained of being chilly and weaker, and of having more abdominal uneasiness after than before. And well he might, for it proved on inquiry, that he was placed in a sitting posture (so that his legs and thighs would escape being wet) during the affusion; his body was not even wiped afterwards: he was allowed to put on his clothes, and then to go to bed without the sheets being removed.

These inconveniences might possibly have been avoided had the above directions been enforced; but in this case they had not been given to the person who superintended.

