# Errata

**Published:** 1804-07-01

**Authors:** 


### ERRATA.

Page 540, line 13, for *nor never will,* read *nor ever will.*
543, 3, from the bottom, for *sensation,* read *cessation.*
538 to 546, inclusive, (running title) for *on opium,* read *on hydrophobia and tetanus,*
547 and 548, for *on opium,* read *on diabetes.*

